# Comparing coronavirus (COVID-19) and climate change perceptions: Implications for support for individual and collective-level policies

**DOI:** 10.3389/fpsyg.2022.996546

**Published:** 2022-10-05

**Authors:** Wouter Poortinga, Briony Latter, Susie Wang

**Affiliations:** ^1^Centre for Climate Change and Social Transformations, Cardiff University, Cardiff, United Kingdom; ^2^School of Psychology, Cardiff University, Cardiff, United Kingdom; ^3^Welsh School of Architecture, Cardiff University, Cardiff, United Kingdom; ^4^Tyndall Centre for Climate Change Research, Cardiff University, Cardiff, United Kingdom; ^5^Climate Outreach, Oxford, United Kingdom; ^6^Faculty of Behavioural and Social Sciences, Department of Social Psychology, University of Groningen, Groningen, Netherlands

**Keywords:** COVID-19, coronavirus, climate change, perceived responsibility, trust, policy support

## Abstract

COVID-19 (coronavirus) and climate change are both global issues that have wide-reaching and serious consequences for human health, the economy, and social outcomes for populations around the world, and both require a combination of systemic governmental policies and community support for action. This paper compares people’s responses to the coronavirus pandemic and climate change in the United Kingdom (UK). A representative survey of the UK population (*n* = 1,518) conducted in November and December 2020 explored public perceptions of (a) personal and government responsibility, (b) efficacy and trust, and (c) support for policies to address the two issues. The results show that, while there are a number of similarities between coronavirus and climate change, major differences exist regarding individual action. In comparison to the coronavirus pandemic, people feel less personal responsibility, think that their own personal actions are less efficacious, and express lower levels of support for (in particular individual-level) policies to address climate change. These findings suggest that experiences from the coronavirus pandemic cannot directly be translated to climate change, and thus that climate change is likely to require different policy responses and framing.

## Introduction

### General background

The COVID-19 (coronavirus) outbreak has had a profound impact on people and communities across the world, sometimes with long-lasting consequences. The outbreak saw unprecedented government and individual action to contain the virus and prolonged periods during which the public were involved in ongoing discussions about acceptable trade-offs to contain the virus and its impacts.

Multiple scholars have drawn parallels between coronavirus and the global climate emergency. Manzanedo and Manning (2020) point to the shared challenges to take early action to prevent higher costs in the long-term, that both have tipping points to be identified and prevented, and that both require global cooperation and action. There are specific lessons from the coronavirus outbreak that can be applied to the climate change emergency, including the need to alert and reassure the public at the same time, and having clear communications from leaders and scientists that both individual and collective action is needed (Ruiu et al., 2020).

However, there are also significant differences between the two issues, including the speed at which the impacts occur and consequently the duration of the actions needed to address them. Risks are emphasised at a personal level for coronavirus compared to different levels of action for climate change, and the risks are also more immediate for coronavirus due to how quickly it spreads (Ruiu et al., 2020). Such differences are recognised by the public as well. A survey of US citizens shows that, while people perceive similarities between the two issues (they are both harmful to public health, politically polarising and global issues), they see the differences in terms of COVID-19 occurring on a shorter time scale, and being natural and medical rather than human-caused (Geiger et al., 2021). Also pertinent is the difference in the complexity and scale of change needed from societies to tackle climate change with no easy solutions. For example, there is no vaccine to inoculate against the risks of climate change (Manzanedo and Manning, 2020).

Even so, there may be lessons to take from how the acceptability of COVID-19 action at both systemic and individual levels could translate to the climate change context, or not. The coronavirus outbreak has shown that governments and other institutions can respond quickly and effectively to counter an urgent global threat, and that society has been largely supportive of these interventions, certainly in the early stages of the pandemic (Howarth et al., 2020). While climate change requires similarly coordinated action at different levels of society, including individuals acting collectively as well as direction and coordination by national and international institutions to enable and facilitate those actions, the perceived roles of individuals, governments and corporations therein are often asymmetric (Capstick et al., 2021); and just as for the response to the coronavirus outbreak, there is a clear need for a social mandate (Howarth et al., 2020).

This study aims to understand UK public perceptions of the two issues and how they compare, particularly for understanding support for individual and collective-level policies. While previous research has already explored the public’s emotional reactions to COVID-19 and climate change and compared judgments of their own personal mitigation behaviours (Geiger et al., 2021), there has been no research that directly compares public support for individual and collective-level policies as well as other key dimensions that are relevant to address COVID-19 and climate change. Below, we outline literature on three core areas that are relevant to how society responds to the two issues: perceived personal and government responsibility, perceived individual efficacy and trust in government, as well as policy support.

### Theoretical background

Responsibility is a key concept in environmental concern, important for understanding when and why people act (Kaiser et al., 1999). In many contexts, it is understood that responsibility is shared, with a simultaneous need for individual action and government policy, for example people acting for the common good and lifestyle interventions from government with regards to climate change (Howarth et al., 2020). Beliefs about where these responsibilities lie can vary widely across different contexts and societal groups (Boto-García and Bucciol, 2020). For example, in the UK, there was a strong emphasis on voluntary behaviour change to contain the coronavirus. Even alongside government mandated lockdowns, personal responsibility to contain the virus was highlighted throughout (Cairney and Wellstead, 2021) and was the ‘dominant frame’ in the UK Prime Minister’s first lockdown announcement (Karyotis et al., 2021). As a result, there was a strong emphasis on individual responsibility, where the blame for the spread of the virus laid upon individuals who failed to comply with social distancing (Karyotis et al., 2021). Research from other European countries shows that people felt a sense of personal responsibility towards mitigating coronavirus and that this was emphasised by policy makers (Farrell et al., 2021; Zimmermann et al., 2021). However, levels of perceived personal responsibility to contain coronavirus differed throughout countries in the Americas, Europe and Asia, with research finding that participants in the Americas agreed with people taking personal responsibility but that this was lower in the South of Europe (Pizarro et al., 2020).

In contrast, people’s sense of individual responsibility for climate change is typically low (Bouman et al., 2020). Research from the US found that people felt a greater sense of responsibility to contain the coronavirus than to tackle climate change (Bostrom et al., 2020), with potential implications for support for policies targeting individuals. In the EU, 63% of Europeans think that national governments should have responsibility for tackling climate change, compared to only 41% stating they should have personal responsibility (European Commission, 2021). These findings are mirrored in the UK, where a higher percentage of the public believe that the government should be responsible for addressing climate change impacts in the UK (34%) compared to the general public (26%) or businesses (19%) having responsibility (Department for Business, Energy and Industrial Strategy, 2021). Fisher et al. (2018, p. 11) reported that people in Britain “do not feel a strong sense of personal responsibility to try to reduce [climate change].” Feelings of responsibility towards climate change can also differ between demographics, with a sense of personal responsibility being higher among older age groups, women and those who are highly educated, and lower among those on the political right (Boto-García and Bucciol, 2020).

As Pohjolainen et al. (2021, p. 3) argue, “ascribing responsibility to oneself implies that one is implicated in fostering climate change, or in seeking solutions to the problem.” However, feeling responsible in itself is not sufficient to motivate action. It also matters whether a person feels able to take action and whether action will lead to certain outcomes, which are often collectively referred to as efficacy beliefs (Bandura, 1995). Research has shown that efficacy beliefs are associated with changes in behaviour related to climate change (Van Valkengoed and Steg, 2019) and may be a key factor in promoting action (Gregersen et al., 2021). Research in Western democracies has found that high personal efficacy led to strong social distancing intentions and compliance with public health advice during the first wave of the coronavirus outbreak (Duong et al., 2021; Jørgensen et al., 2021). However, personal efficacy tends to be lower for climate change than for coronavirus (Bostrom et al., 2020; Howarth et al., 2020). While a high percentage (71%) of the UK public feel they can take personal action to address climate change, some groups feel they can do so more than others. For example, women generally feeling more able to make personal changes than men (Department for Business, Energy and Industrial Strategy, 2021). Salomon et al. (2017) found that feelings of helplessness negatively affect people’s ability to take steps to address climate change. People have lower intentions to act on climate change if they feel their actions have no meaningful impact. There are also implications for government policy, for example, where higher personal efficacy can predict greater support for environmental policies (Wolters et al., 2020).

In addition to personal efficacy, the extent to which the public trusts the ability of the government to act effectively is an important element of both individual action and policy support (Lorenzoni and Pidgeon, 2006). In respect of the pandemic, policy acceptance (and hence efficacy) has been dependent in part on public trust in government’s ability to organise effective policies and to consider how they will impact people in response to the pandemic (Cairney and Wellstead, 2021). It has been argued that both social trust (between citizens) and political trust (between people and their governments) are critical to ensure that government recommendations to contain the coronavirus are acted upon; government trust to ensure that people feel that the recommendations will be beneficial, and social trust to ensure confidence that others will follow government recommendations as well (Harring et al., 2021). During the initial coronavirus lockdowns, people’s trust in government increased in some Western European countries where lockdown measures were seen as necessary and led to an increase in “support for the status quo decision makers, institutions and regimes” (Bol et al., 2021, p. 498). Yet, public trust, which was initially high, had decreased by the time the initial lockdown had lifted (Cairney and Wellstead, 2021). Research with a small sample of the UK public found that there was also a lack of trust in government communication about the personal actions people should take to limit the spread of coronavirus (Williams et al., 2020).

Trust is equally important for climate change policies because the kind of mitigation policies that may impact individuals’ lives in restrictive ways requires trust between citizens and the government (Klenert et al., 2020). Trust in government has been shown to be associated with more positive climate policy attitudes (Kulin and Johansson Sevä, 2021), and when the government is perceived as trustworthy, people’s adherence to voluntary and mandatory measures is higher (Schmelz, 2021). If there is a lack of trust, or if governments are seen to be saying one thing and doing another, then there can be a reduction in cooperation (Mintrom and O’Connor, 2020). This not only applies to policy support and compliance, but also to the willingness to take individual action. The public may consider individual action futile if governments appear to absolve their duty in meeting collective interests of society. In contrast, a culture of trust, coupled with high concern about the climate, can foster feelings of personal responsibility (Bodor et al., 2020) and lead to individual action (Smith and Mayer, 2018).

Meeting ambitious targets to keep climate change within a 1.5°C temperature increase requires substantial changes to the way we live and organise our societies (Capstick et al., 2021). These changes need to be accelerated by public policy in order to succeed at mitigating climate change (Roberts et al., 2018). However, meaningful change can only happen with sufficient social mandate from the general public (Howarth et al., 2020). Opposition to policies may lead to delays or cancellation, which can be costly and provide suboptimal outcomes (Klenert et al., 2020). Public perception can also influence policymakers to enact more ambitious measures (Klenert et al., 2020). Strong public support for policies to address societal risks can bring about a shift in social norms, and greater demand for policy action. High levels of support were seen for coronavirus containment measures in the UK, with 82% of the public agreeing that actions taken by the government were necessary (Collignon et al., 2021). Such support may have played a part in enabling the implementation of unprecedented policies with far-reaching consequences for individuals, communities and the economy.

Societal threats, such as climate change, may be contained through a range of policy options that may target both individuals and businesses, including limiting what people can do through regulation, issuing fines and providing financial support and information. Support for measures may vary according to their policy attributes (e.g., Swim and Geiger, 2021), individual factors such as perceptions of responsibility, fairness, and trust in government (e.g., Bostrom et al., 2020; Kulin and Johansson Sevä, 2021; Bergquist et al., 2022), and context, for example, where infringements on personal freedoms may be seen differently depending on the issue (e.g., Klenert et al., 2020).

We have seen examples of different types of policy to contain the coronavirus. For example, limits on personal activity have been implemented in almost every country through restrictions on physical proximity, social gatherings, curfews, and more (Hale et al., 2021); fines have been used to enforce compliance (Wadvalla 2020; Mazerolle and Ransley, 2021; Politis et al., 2021); and governments have implemented financial support measures for businesses, entrepreneurs, and the population in general to help cope with the effect of the pandemic, such as through job retention or furlough schemes (Mayhew and Anand, 2020). Finally, public health information has been deployed to varying degrees in all countries dealing with coronavirus to provide guidance on how to reduce the spread of the virus (e.g., “Stay Home. Protect the NHS. Save Lives” in the UK, or the ‘3Cs’ in Japan, which referred to “Closed Spaces, Crowded Places and Close Contact,” Barnes, 2021). The measures that have been implemented have led to fast and wide-ranging changes for individuals and at a systemic level (Hochachka, 2020), with international research showing a high percentage of people self-reporting that they have been following the coronavirus measures (YouGov, 2021).

The same types of measures could potentially help to address climate change, but may be more controversial in that context. Research shows that curbing or banning high-carbon behaviours is seen as desirable in theory, but less so when people are directly affected. Although an overwhelming majority of the UK population think we should ‘probably’ or ‘definitely’ limit how much flying people do (Steentjes et al., 2021), there is a lower willingness to accept policies that restrict individual travel (Higham et al., 2016; Kantenbacher et al., 2018). There is also high public support in the UK for reducing meat consumption (Whitmarsh, 2020), but other research has shown that, out of a choice between whether people would be most willing to ban non-essential flying, meat, or petrol and diesel vehicles, only 6% of people would choose a meat ban (Ash and Zimmermann, 2020). Other impactful measures, such as a ban on petrol and diesel cars receive less than majority support (30% for those aged 16–29 and 21% for those aged 50–69; Ash and Zimmermann, 2020). Support for the use of fines as punishment for undesirable behaviours is also mixed, with fines for low-cost individual behaviours such as littering being seen as more acceptable than those for high-cost behaviours such as car use (de Groot and Schuitema, 2012). Support for fines for companies or corporations is higher. Stead (2018) found that making companies pay large fines is seen by citizens as an effective way of dealing with pollution. Swim and Geiger (2021) reported that policies targeting businesses are more supported than policies targeting individuals, in particular those that use disincentives, such as a carbon tax.

Other, more moderate, measures tend to receive greater support. For instance, financial support and the provision of information tend to be less controversial climate change policies. In general, people prefer policies that use incentives (‘pull’ measures) rather than disincentives (‘push’ measures), especially when they are targeted at individuals (Drews and van den Bergh, 2016; Swim and Geiger, 2021). Similarly, using public money to subsidise renewable energy is a policy which has high support from the public in many European countries, especially in comparison to taxes and bans (Poortinga et al., 2018; Davidovic and Harring, 2020). Awareness or information-based policies are also seen as important by the public. For example, education and good government communication is seen as a priority by a majority of Climate Assembly UK members in relation to changes required in the home to tackle climate change (Climate Assembly UK, 2020). However, these policies, while popular, cannot be relied upon solely to limit climate change, and there is a need to understand the mechanisms that bring about public support for more costly policies as well. There is a question whether policies and practices implemented during the pandemic can translate to climate change and other risks, and in how these experiences have impacted people’s willingness to consider government intervention when it comes to restrictions on their personal freedoms and economic outcomes.

### This study

Building on an emerging research field with limited empirical research (Bostrom et al., 2020; Ecker et al., 2020; Geiger et al., 2021), this paper seeks to understand similarities and differences in people’s responses to the coronavirus outbreak and climate change in the UK, and the lessons these hold for what may or may not be possible in terms of public engagement and action on climate change. More specifically, it will examine perceptions of responsibility (both personal and governmental), the perceived effectiveness of individual action and trust in government, as well as the acceptability of different types of policies to address the two issues. The existing literature outlined above suggests that there may be some differences and similarities between the two issues. First, it appears that people’s sense of responsibility is higher for coronavirus than for climate change, but that perceived government responsibility is high for both coronavirus and climate change. Second, personal efficacy appears to be higher for coronavirus than for climate change. It is more difficult to divine the differences regarding government trust due to limited existing research. It could however be expected that trust in government is low for both issues, with growing perceptions of UK government incompetence regarding the handling of the coronavirus crisis (Cairney and Wellstead, 2021) and low trust in the government to take effective action (Steentjes et al., 2021). Third, a general pattern can be expected of coercive ‘push’ measures (e.g., regulation, fines) being less supported that non-coercive ‘pull’ measures, such as subsidies, financial support and information provision (Drews and Van den Bergh, 2016) and policies aimed at individuals being less supported than policies aimed businesses, in particular those that use disincentives (Swim and Geiger, 2021).

There are no firm expectations regarding the differences between coronavirus and climate change, but it appears that people have been more accepting of restrictions and fines to contain the outbreak of the coronavirus. It can therefore be anticipated that coercive measures are more supported to deal with coronavirus than with climate change. Note that these are general expectations derived from the literature review rather than firm hypotheses that will be tested formally.

Existing research has largely focused on either coronavirus *or* climate change, making it difficult to draw clear conclusions. *First*, the paper compares perceptions of personal and government responsibility, perceived efficacy and trust and support for different types of policies to address coronavirus and climate change, using matched policies. *Second*, it explores the role of individual-level variables in coronavirus-and climate change-related perceptions and policy support. The individual-level variables include the socio-demographics of gender and age, and political orientation, (general) trust in government and worry about coronavirus and climate change. Gender, political orientation, trust and worry (or concern) are consistent predictors of climate change views (Weber, 2010, 2016), and age was communicated as a key risk factor for vulnerability to coronavirus (World Health Organization, 2020). *Third*, the paper assesses the extent to which the individual-level variables can explain differences in coronavirus-and climate change-related perceptions and policy support.

This paper contributes towards addressing existing gaps in research by directly comparing the public’s responses to coronavirus and climate change along these three dimensions above, and exploring implications for future engagement and action on climate change. It also has practical implications for policymakers in terms of the types of responses and framing used for coronavirus and climate change that can inform future policy development. The main limitations of the research relate to the temporal context and location – it is UK-based and therefore may not be applicable to other countries, and the survey was conducted at a particular stage of the pandemic which may have impacted the responses.

## Materials and methods

### Survey and sample

The study uses data that were collected as part of a project on engagement with climate change at the start of the second wave of the coronavirus outbreak in the UK (Shaw and Wang, 2021). The study was designed to develop and test narratives for climate engagement and to explore similarities and differences in UK public perceptions of coronavirus and climate change. The project consisted of several elements, including an expert advisory board, stakeholder panel, community panel, and a nationally-representative quantitative survey. The advisory board and stakeholder roundtable discussion groups were used to inform the design and content of the survey.^1^

Following this, a nationally representative survey of British residents (*N* = 1,518) was conducted online from 19 November to 12 December 2020 by DJS Research, a market research company. The survey approximated representation through quotas for gender, age, region, and ethnicity (Office for National Statistics, 2019, 2020; see Table 1 for details). The survey extended previous research by systematically comparing perceptions of (a) personal and government responsibility, (b) efficacy and trust, and (c) support for policies to address the two issues. The survey also used a novel approach to understand the trade-offs between hazards reduction, economic impact and personal freedom that people are willing to make (Shaw and Wang, 2021), and further included two ‘test’ narratives: one exploring respondents’ sense of agency and another exploring the potential for health messaging to which respondents could respond (Shaw and Wang, 2021). Ethical approval was granted by the School of Psychology Research Ethics Committee at Cardiff University (Shaw and Wang, 2021).

**Table 1:** Characteristics of the sample (*N* = 1,518).

	Category	*N*	% sample	% population*
Gender	Female	773	52.3	49.4
	Male	734	47.2	50.6
	Prefer not to say/prefer to be described another way	8	<1.0	–
Age	18–24	103	6.7	10.7
	25–49	579	38.1	41.4
	50–64	457	30.1	24.3
	65+	369	24.3	23.5
	Prefer not to say	10	<1	–
Region	East Anglia	126	8.3	9
	East Midlands	102	6.7	7
	London	212	14.0	13
	North East	58	3.8	4
	Northern Ireland	39	2.6	3
	North West	156	10.3	11
	Scotland	214	14.1	8
	South East	189	12.5	14
	South West	107	7.0	8
	Wales	69	4.5	5
	West Midlands	126	8.3	9
	Yorkshire and Humberside	120	7.9	8
Ethnicity	Arab	3	0.2	0.4
	Asian	119	7.9	7.5
	Black	43	2.8	3.3
	Mixed ethnic background	40	2.6	2.2
	Prefer not to say	14	0.9	–
	White	1,293	85.2	86
	Missing	6	0.4	–

* Gender, age and region data obtained from the Office for National Statistics (2019). Ethnicity data obtained from the (2020). Age percentages are scaled based on the over 18 population. Population statistics for non-binary gender unavailable.

### Survey questions and measures

Participants were asked a series of matched questions about preventing coronavirus from spreading or climate change from worsening. The main variables used for the analyses in this paper addressed perceived personal and government responsibility, trust in government, perceived efficacy of personal actions, support for different policies to address the two issues, and a number of control variables. The questions followed a fixed order, but the coronavirus and climate change sections were counter balanced, with 50% of the respondents starting with the questions about coronavirus and 50% of the respondents starting with the questions about climate change. The order of the policies was randomised for both sections. The full questionnaire is available in the Open Science Framework repository, including measures that have not been used in this paper (https://osf.io/2x3m6/).

#### Perceived personal and government responsibility

Respondents were asked to what extent they feel it is their own personal responsibility and to what extent they think it is the government’s responsibility to try to prevent [coronavirus from spreading/climate change from worsening]. Respondents used an end-labelled 11-point scale, ranging from 0 (not at all) to 10 (a great deal) to answer these questions. The items were adapted from the personal responsibility question developed for the European Social Survey (Poortinga et al., 2018) so that the phrasing was similar across the four items.

#### Perceived personal efficacy

Respondents were asked “To what extent do you feel that your own personal actions can help prevent [coronavirus from spreading/climate change from worsening]” in order to measure respondents’ perceived efficacy (also known as ‘outcome expectancy’, Gregersen et al., 2021). A similar end-labelled 11-point scale, ranging from 0 (not at all) to 10 (a great deal), was used for this question. The perceived personal efficacy items were adapted from efficacy questions developed for the European Social Survey (Poortinga et al., 2018). The items were rephrased so that the efficacy applied to preventing coronavirus from spreading/climate change from worsening in line with the responsibility questions.

#### Trust in (the efficacy of) government

Trust in government was measured with the items “To what extent do you trust the UK government to take effective action to help prevent [coronavirus from spreading/climate change from worsening].” The response scale ranged from 0 (not at all) to 10 (a great deal) with only the end points labelled. The items were newly developed for the purpose of the study so that the phrasing would align with the responsibility and efficacy questions.

#### Policy support

Respondents were asked to what extent they support or oppose a range of policies that could be used to contain coronavirus and climate change. The different policy options covered limiting activity through regulation, financial incentives (fines and financial support), and information provision; and focused on either individuals or businesses (see Table 2). These measures have been put in place in numerous countries for coronavirus (International Monetary Fund, 2021) and some have been enacted for climate change, such as fines and information provision (Department for Levelling Up, Housing and Communities and Ministry of Housing, Communities and Local Government, 2019; Environment Agency, 2022). Policy support was measured by asking respondents to answer “Would you support or oppose the following measures to prevent [coronavirus from spreading/climate change from worsening]” on a 5-point scale from 1 (strongly oppose) to 5 (strongly support). The items were developed for the specific purpose of the study. The items were selected and phrased so that they were comparable across the two issues.

*Predictor variables*: The survey further asked respondents for their gender and age, as well as their worry about climate change and coronavirus, political orientation and general trust in the government. The age variable was centred on its mean and expressed in 10-year deviations from that mean. *Worry about climate change and coronavirus* was measured by asking respondents “How worried are you about [climate change/coronavirus]?,” with a response scale ranging from 1 (not at all worried) to 5 (extremely worried), adapted from Poortinga et al. (2018). At the time of the survey, which was conducted at the start of the second wave of the coronavirus outbreak, worry about the coronavirus (*M* = 3.70, SD = 1.10) was higher than about climate change (*M* = 3.44, SD = 1.15). *Political orientation* (*M* = 5.16, SD = 2.10) involved self-placement on an end-labelled 11-point scale ranging from 0 (left) to 10 (right). The question was sourced from the European Social Survey, 2016. *General trust in government* (*M* = 4.34, SD = 2.73) was measured by asking respondents to answer “Generally speaking, would you say you trust or distrust the UK government” on an end-labelled 11-point scale from 0 (completely distrust) to 10 (completely trust). This item was newly developed for the current study. Both the political orientation and general trust in government variables were standardised by calculating *Z* scores.

**Table 2:** Matched policy measures to address coronavirus and climate change.

Type of policy	Individual-level	Business-level
Limit activity	Limit the activity of individuals (e.g., banning unnecessary air travel)	Limit the activity of businesses (e.g., closing high-polluting/non-essential businesses)
Fines	Fining individuals who do not uphold coronavirus/climate change regulations	Fining businesses that do not uphold coronavirus/climate change regulations
Financial support	Financial incentives encouraging people to follow coronavirus/climate change regulations	Financial support for businesses to deal with the costs of coronavirus/climate change policies
Information	Providing information to individuals about what they can do to prevent coronavirus/ climate change from spreading	Providing information to businesses about what they can do to prevent coronavirus/ climate change from spreading

Additional (dummy) variables were created to indicate whether measures were about (i) *coronavirus* versus *climate change*, (ii) *personal* versus *government responsibility*, and (iii) *perceived efficacy* versus *trust*. Further dummy variables were used to indicate whether the policies were aimed at (iv) *individuals* versus *businesses*, and to indicate (v) the *type of policy* (i.e., limiting activity, fines, and financial support versus information).

### Statistical analysis

All analyses were performed in R Studio v1.3.1073, a development environment for the R statistical software (v4.02). Several R packages were used, including the *tidyverse* (Wickham et al., 2019) and *lme4* (Bates et al., 2015) packages. This study used a multilevel modelling approach to analyse the data, with responses to questions (Level 1) being nested within participants (Level 2). This means that the responses to the different questions are considered repeated measures provided by the participants. This multilevel repeated-measures approach was taken to apportion variance specific to the different measures and variance common to the individuals taking part in the study. This approach allows for (cross-level) interactions between measure-specific and individual-level characteristics.

Three sets of multilevel models were constructed for *perceived personal and government responsibility* (Models A), *perceived efficacy and trust* (Models B), and *policy support* (Models C), respectively. The dataset was converted into long format whereby each row represented a measurement occasion for each of the three sets of repeated measures multilevel models. For example, for the personal versus government responsibility analyses, there were four rows for each participant representing their ratings regarding personal and government responsibility for coronavirus or climate change, respectively.

Each of the three sets of analyses consisted of four subsequent models. First, null models, without any predictors, were created (Models A0, B0, and C0). These models were used to determine the intraclass correlation (ICC). The ICC reflects the proportion of the variance that is common across rather than specific to the different measures (and thus here reflects variance that can be attributed to the individual). The ICC indicates the extent to which a multilevel approach is appropriate. Second, analyses were conducted for perceived personal responsibility versus government responsibility (Models A1), perceived efficacy and trust (Models B1), and support for a range of policies (Models C1), respectively. These models (Step 1) included the measure-specific dummy variables of *coronavirus* versus *climate change*, as well as the dummy variables of *personal* versus *government responsibilit*y (Models A1), *perceived efficacy* versus *trust* (Models B1) or policies aimed at *individuals* versus *businesses* (Models C1). Interaction terms were added in Step 2. Third, analyses were conducted separately for perceived personal responsibility and perceived government responsibility (Models A2), for personal efficacy and government trust (Models B2), and for policies aimed at individuals and policies aimed at businesses (Models C2), respectively. These models included the *coronavirus* versus *climate change* dummy and the individual-level variables of gender, age, political orientation, general trust in government, and worry about climate change and coronavirus. Fourth, Models A3, B3 and C3 added cross-level interactions between the *coronavirus* versus *climate change* dummy and the individual-level variables of gender, age, etc.

Further analyses were conducted for support for climate change and coronavirus policies aimed at individuals and businesses separately (Models C4). These additional models were used to explore in more detail individual differences in support for the specific policies. Models C4 included a measure-specific variable indicating *policy type* and the individual-level variables of gender, age, political orientations, trust in government, and worry about climate change and coronavirus; as well as cross-level interactions between policy type and the individual-level variables. The results of these analyses are not reported in detail in the text, but are available in the supplementary information, Supplementary Table SI6.

## Results

### Perceived personal and government responsibility

**Figure 1:**
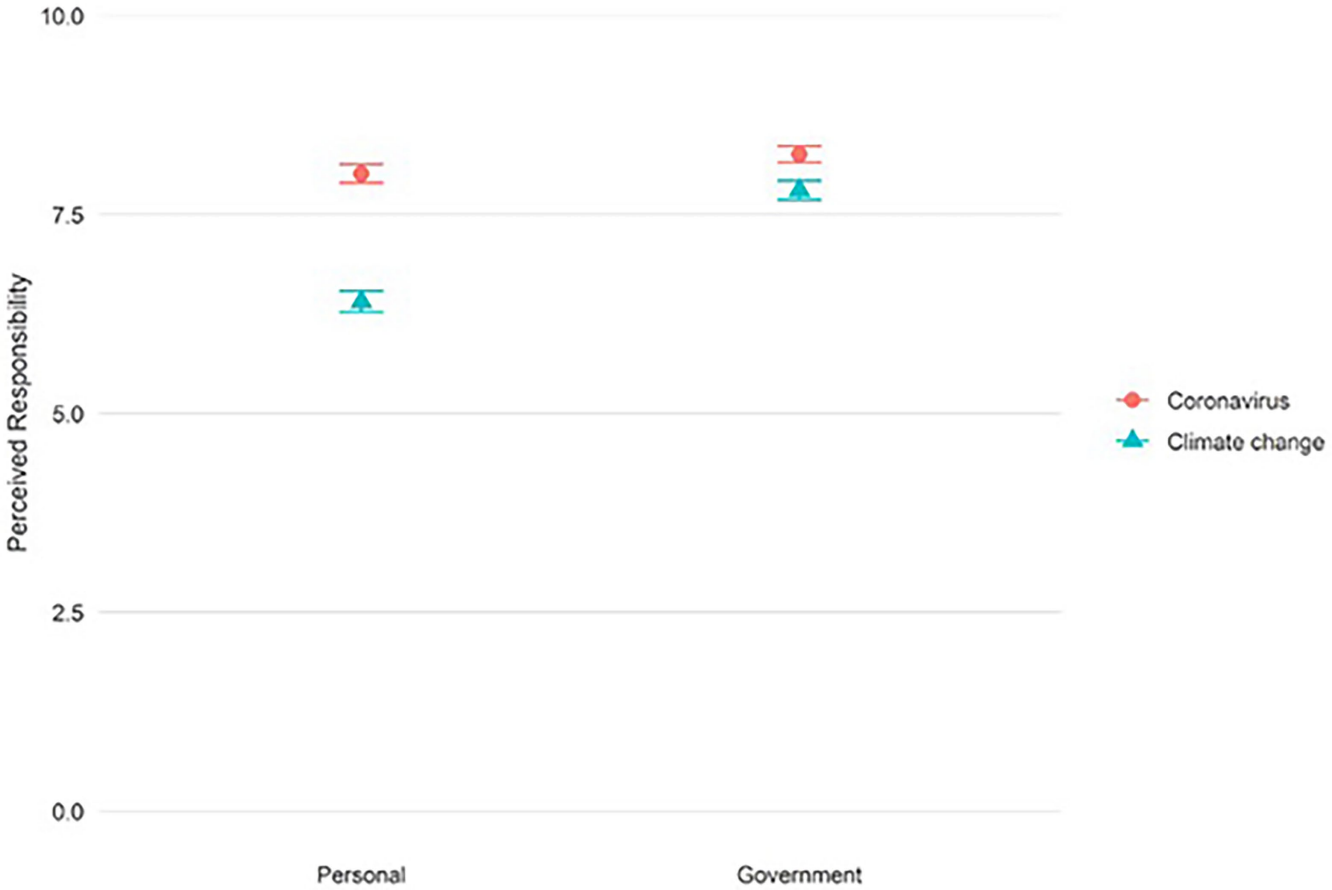
Mean scores for personal responsibility and government responsibility to try to prevent coronavirus from spreading (circle) and climate change from worsening (triangle). Error bars reflect the 95% confidence intervals (also see Supplementary Table SI1).

The first set of analyses (Models A) examined public views on perceived personal and government responsibility regarding coronavirus and climate change. Figure 1 shows that respondents think that both the government (*M* = 8.26, SD = 2.10) and they themselves personally (*M* = 8.01, SD = 2.33) have a responsibility to stop coronavirus from spreading (also see supplementary information, Supplementary Table SI1). While respondents also ascribe a high level of responsibility to the government to try to reduce the risk of climate change (*M* = 7.81, SD = 2.39), they feel less personally responsible to do so (*M* = 6.41, SD = 2.67). The score was however still above the scale midpoint of 5, suggesting that people still think they have some personal responsibility to address climate change, only less so than for coronavirus.

Model A0 shows that 40% (ICC = 0.400) of the variance can be found at the individual level, indicating there is substantial shared variance across the four responsibility measures. The relatively high shared variance shows that the responsibility measures are not independent and thus that a multilevel repeated measures approach is appropriate. Results from Model A1 (Step 1) show that there is a significant main effect for coronavirus versus climate change, with people ascribing lower levels of personal and government responsibility to deal with climate change than with coronavirus (*B* = −1.030, 95% CI [−1.119, −0.941]). There is also a significant main effect for personal versus government responsibility, with overall higher government responsibility than personal responsibility ratings (*B* = 0.820, 95% CI [0.731, 0.909]). A significant interaction effect in Step 2 indicates a larger difference in perceived personal and government responsibility for climate change than for coronavirus (*B* = 1.155, 95% CI [0.981, 1.330]). This interaction reflects the lower perceived personal responsibility for climate change than for coronavirus (see Figure 1). The full results for Model A1 are provided in the supplementary information, Supplementary Table SI2.

Table 3 shows the results for Models A2 and A3. Perceived personal responsibility was predicted by gender, age, political orientation, general trust in government, and worry about both climate change and coronavirus (Model A2). There were a number of significant interactions (Model A3), with age and worry about coronavirus being weaker predictors of perceived personal responsibility to deal with climate change than of perceived personal responsibility to deal with coronavirus. Worry about climate change was a stronger predictor of perceived personal responsibility to deal with climate change than of perceived personal responsibility to deal with coronavirus. Non-significant interactions suggest that gender, political orientation and general trust in government are all equally strong predictors of perceived personal responsibility to address coronavirus and of perceived personal responsibility to address climate change.

**Table 3:** Perceived personal and government responsibility (Models A2 and A3).

	Personal responsibility		Government responsibility	
	Model A2 B (95%CI)	Model A3 B (95%CI)	Model A2 B (95%CI)	Model A3 B (95%CI)
*Fixed effects*				
Constant	7.313^***^ (7.169, 7.457)	7.451^***^ (7.296, 7.606)	7.845^***^ (7.711, 7.979)	7.978^***^ (7.834, 8.122)
Climate change (reference: Coronavirus)	−1.603^***^ (−1.737, −1.469)	−1.879^***^ (−2.057, −1.702)	−0.449^***^ (−0.566, −0.333)	−0.716^***^ (−0.874, −0.558)
Female (reference: Male)	0.296^***^ (0.122, 0.471)	0.285^***^ (0.072, 0.497)	−0.031 (−0.196, 0.134)	−0.064 (−0.261, 0.134)
Age	0.119^***^ (0.063, 0.175)	0.227^***^ (0.159, 0.295)	0.059^**^ (0.006, 0.113)	0.100^***^ (0.036, 0.164)
Political orientation	−0.129^***^ (−0.226, −0.032)	−0.120^**^ (−0.238, −0.001)	−0.346^***^ (−0.438, −0.254)	−0.325^***^ (−0.435, −0.214)
General trust in government	0.266^***^ (0.172, 0.360)	0.291^***^ (0.176, 0.406)	0.083 (−0.007, 0.172)	0.085 (−0.022, 0.192)
Climate change worry	0.894^***^ (0.812, 0.975)	0.350^***^ (0.250, 0.449)	0.725^***^ (0.648, 0.803)	0.277^***^ (0.184, 0.369)
Coronavirus worry	0.417^***^ (0.335, 0.500)	0.738^***^ (0.637, 0.840)	0.314^***^ (0.235, 0.393)	0.558^***^ (0.464, 0.652)
Climate change × Gender		0.023 (−0.220, 0.266)		0.065 (−0.152, 0.282)
Climate change × Age		−0.217^***^ (−0.295, −0.138)		−0.081^**^ (−0.151, −0.011)
Climate change × Political orientation		−0.019 (−0.155, 0.117)		−0.043 (−0.164, 0.078)
Climate change × General trust in government		−0.050 (−0.181, 0.081)		−0.005 (−0.122, 0.112)
Climate change × Climate change worry		1.088^***^ (0.974, 1.202)		0.897^***^ (0.796, 0.999)
Climate change × Coronavirus worry		−0.642^***^ (−0.758, −0.526)		−0.489^***^ (−0.592, −0.386)
*Random effects*	σ2 (SD)	σ2 (SD)	σ2 (SD)	σ2 (SD)
Level 2 (individual)	1.018 (1.009)	1.428 (1.195)	1.165 (1.079)	1.419 (1.191)
Level 1 (measure)	3.524 (1.877)	2.704 (1.644)	2.656 (1.630)	2.149 (1.466)
*Model Fit*				
AIC	13,068	12,680	12,451	12,142
BIC	13,122	12,764	12,506	12,226

Analyses based on 3,010 observations within 1,505 individuals.

** *p* < 0.05 and

*** *p* < 0.01.

The right-hand columns of Table 3 show that perceived government responsibility was predicted by age, political orientation, and worry about both climate change and coronavirus (Model A2). There were a number of significant interactions (Model A3), showing that age and worry about coronavirus are weaker predictors of perceived government responsibility to deal with climate change than of perceived government responsibility to deal with coronavirus. Worry about climate change was a stronger predictor of perceived government responsibility to deal with climate change than of perceived government responsibility to deal with coronavirus. A non-significant interaction suggests that political orientation is an equally strong predictor of perceived government responsibility to address coronavirus and of perceived personal responsibility to address climate change.

### Perceived personal efficacy and trust in (the efficacy of) government

**Figure 2:**
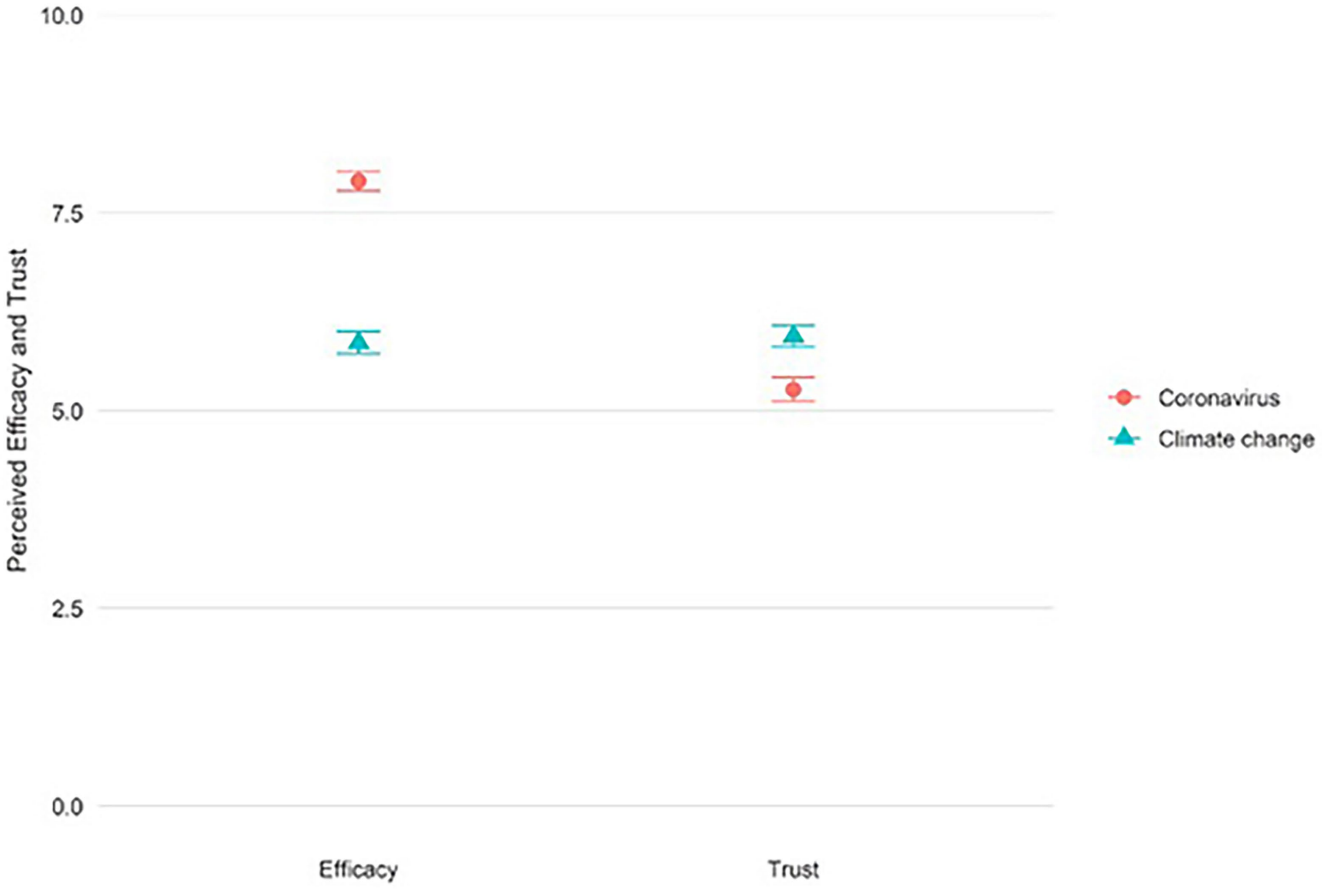
Mean scores for efficacy of personal actions, and trust in government to try to prevent coronavirus from spreading (circle) and climate change from worsening (triangle). Error bars reflect the 95% confidence intervals (also see Supplementary Table SI1).

Figure 2 shows the comparison of mean responses to perceived personal efficacy and trust, for both coronavirus and climate change. Respondents thought their own personal actions were highly effective in preventing coronavirus from spreading (*M* = 7.90, SD = 2.40), but less so in preventing climate change from worsening (*M* = 5.86, SD = 2.78). Respondents trusted the government slightly more in relation to climate change (*M* = 5.95, SD = 2.78) than in relation to coronavirus (*M* = 5.27, SD = 3.02). Trust scores were in both cases only slightly higher than the scale midpoint of 5, showing that, on average, there is a moderate level of trust for the two issues.

Model B0 shows that 27% of the variance can be found at the individual level (ICC = 0.274), reflecting the extent to which the efficacy and trust judgments can be attributed to the individual rather than the specific measures. This suggests that also here a multilevel repeated measures approach is appropriate. Model B1 (Step 1) shows that on average respondents have lower levels of perceived efficacy and trust in relation to climate change than in relation to coronavirus (*B* = −0.682, 95% CI [−0.800, −0.564]) and that trust judgments are generally lower than efficacy ratings (*B* = −1.275, 95% CI [−1.393, −1.157]). A significant interaction in Step 2 indicates that the difference between perceived personal efficacy and trust was smaller for climate change than for coronavirus (*B* = 2.719, 95% CI [2.496, 2.942]). This result reflects that personal efficacy is higher for coronavirus than for climate change (see Figure 2). The full results for Model B1 are provided in the supplementary information, Supplementary Table SI3.

Table 4 shows the results for the subsequent models for perceived personal efficacy and trust, respectively (Models B2 and B3). The results show that women, respondents with higher levels of general trust in government, and those who are more worried about both climate change and coronavirus are more likely to think their own personal actions can help to address the two issues (Model B2). Interactions show that worry about climate change is a stronger predictor of perceived personal efficacy in relation to climate change, whereas worry about coronavirus is a stronger predictor of perceived personal efficacy in relation to coronavirus (Model B3). While age was not a significant predictor of personal efficacy overall (see Model B2), Model B3 shows that age was positively associated with personal efficacy in relation to coronavirus but negatively so with personal efficacy in relation to climate change.

**Table 4:** Perceived personal efficacy and government trust (Models B2 and B3).

	Personal efficacy		Government trust	
	Model B2 B (95%CI)	Model B3 B (95%CI)	Model B2 B (95%CI)	Model B3 B (95%CI)
*Fixed effects*				
Constant	7.221^***^ (7.066, 7.375)	7.343^***^ (7.175, 7.511)	5.056^***^ (4.915, 5.196)	5.062^***^ (4.909, 5.216)
Climate change (reference: Coronavirus)	−2.041^***^ (−2.180, −1.903)	−2.286^***^ (−2.478, −2.093)	0.683^***^ (0.571, 0.795)	0.670^***^ (0.504, 0.835)
Female (reference: Male)	0.327^***^ (0.138, 0.516)	0.284^**^ (0.054, 0.515)	0.152 (−0.025, 0.328)	0.152 (−0.058, 0.362)
Age	−0.010 (−0.071, 0.051)	0.124^***^ (0.050, 0.199)	−0.063^**^ (−0.120, −0.006)	−0.027 (−0.094, 0.041)
Political orientation	−0.047 (−0.152, 0.059)	−0.108 (−0.236, 0.021)	0.103^**^ (0.004, 0.201)	0.105 (−0.012, 0.223)
General trust in government	0.411^***^ (0.309, 0.513)	0.465^***^ (0.341, 0.590)	2.027^***^ (1.932, 2.122)	2.207^***^ (2.093, 2.320)
Climate change worry	0.805^***^ (0.717, 0.894)	0.372^***^ (0.264, 0.480)	0.132^***^ (0.049, 0.215)	0.078 (−0.021, 0.176)
Coronavirus worry	0.435^***^ (0.345, 0.525)	0.707^***^ (0.598, 0.817)	0.215^***^ (0.131, 0.299)	0.265^***^ (0.165, 0.364)
Climate change × Gender		0.086 (−0.178, 0.350)		−0.0005 (−0.227, 0.226)
Climate change × Age		−0.268^***^ (−0.353, −0.183)		−0.073^**^ (−0.146, −0.0001)
Climate change × Political orientation		0.122 (−0.025, 0.269)		−0.006 (−0.132, 0.121)
Climate change × General trust in government		−0.108 (−0.250, 0.035)		−0.360^***^ (−0.482, −0.237)
Climate change × Climate change worry		0.866^***^ (0.743, 0.990)		0.108^**^ (0.002, 0.215)
Climate change × Coronavirus worry		−0.545^***^ (−0.671, −0.420)		−0.099 (−0.206, 0.009)
*Random effects*	σ2 (SD)	σ2 (SD)	σ2 (SD)	σ2 (SD)
Level 2 (individual)	1.393 (1.180)	1.681 (1.297)	1.632 (1.277)	1.679 (1.296)
Level 1 (measure)	3.757 (1.938)	3.181 (1.783)	2.448 (1.565)	2.354 (1.534)
*Model fit*				
AIC	13,440	13,202	13,684	13,668
BIC	13,494	13,286	13,738	13,752

Analyses based on 3,010 observations within 1,505 individuals.

** *p* < 0.05 and

*** *p* < 0.01.

The right-hand columns of Table 4 show that government trust in relation to the two topics was predicted by gender, age, political orientation, general trust in government, and worry about both coronavirus and climate change (Model B2). Interactions in Model B3 show that age is only a negative predictor of government trust in relation to climate change (and not in relation to coronavirus). They further show that general trust in the government is a stronger predictor of government trust in relation to coronavirus than in relation to climate change; worry about climate change is a stronger predictor of government trust in relation to climate change; and worry about coronavirus is a stronger predictor of government trust in relation to coronavirus. A non-significant interaction shows that political orientation is an equally strong predictor of government trust in relation to coronavirus and of government trust in relation to climate change.

### Policy support

**Figure 3:**
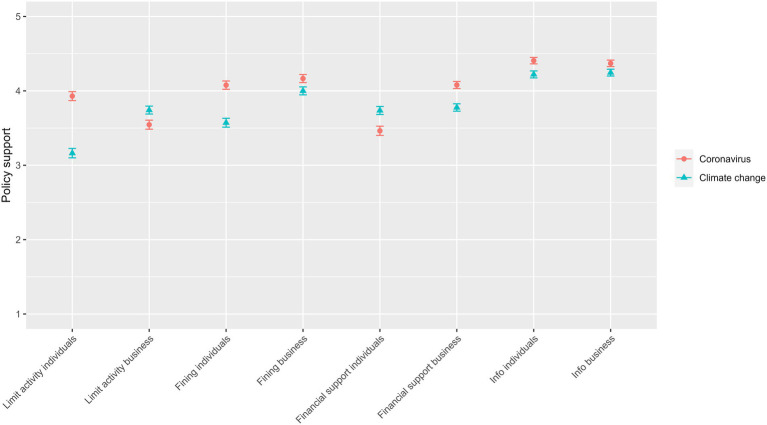
Support for different types of policies to address coronavirus and climate change.

Figure 3 shows policy support for different types of policies to address coronavirus and climate change (also see supporting information, Supplementary Table SI4). It shows that support for all policies was above the midpoint of 3, meaning that on average, none of the suggested policies were ‘opposed’ as such by respondents. The highest levels of support were for information policies, informing individuals and businesses about what they can do to contain coronavirus (*M* = 4.41, SD = 0.86 and *M* = 4.37, SD = 0.86, for individuals and businesses respectively) and climate change (*M* = 4.22, SD = 0.93 and *M* = 4.25, SD = 0.91, for individuals and businesses respectively). Levels of support were also high for fining individuals and businesses who do not uphold coronavirus regulations (*M* = 4.08, SD = 1.11 and *M* = 4.17, SD = 1.08, for individuals and businesses respectively), although slightly lower for climate change (*M* = 3.57, SD = 1.19 and *M* = 4.00, SD = 1.07, for individuals and businesses respectively). Support for financial support to deal with the costs of coronavirus (*M* = 3.46, SD = 1.23 and *M* = 4.08, SD = 0.94, for individuals and businesses respectively) and climate change (*M* = 3.74, SD = 1.07 and *M* = 3.78, SD = 0.99, for individuals and businesses respectively) regulations were slightly lower. The lowest levels of support were found for limiting activity to stop the spread of coronavirus (*M* = 3.93, SD = 1.19 and *M* = 3.55, SD = 1.21, for individuals and businesses respectively) and in particular to stop climate change worsening (*M* = 3.16, SD = 1.25 and *M* = 3.74, SD = 1.06, for individuals and businesses respectively).

Model C0 found that 33% of the variance in policy support is shared across the 16 coronavirus and climate change policies (ICC = 0.329), again showing that a multilevel repeated measures approach is appropriate. Results from Model C1 (Step 1) show that support was lower for climate change policies than for coronavirus policies (*B* = −0.197, 95% CI [−0.220, −0.175]), and lower for policies targeting individuals than for those targeting businesses (*B* = −0.169, 95% CI [−0.192, −0.147]). There was also a significant interaction effect in Step 2, indicative of lower support for individual policies to address climate change relative to those to address coronavirus (*B* = −0.197, 95% CI [−0.243, −0.151]). The full results for Model C1 are provided in the supplementary information, Supplementary Table SI5.

Interactions in Model C3 show that, for policies aimed at individuals, age is a significant predictor of support for coronavirus policies, but not of support for climate change policies (see Table 5). Interactions show that worry about climate change is a stronger predictor of support for climate change policies aimed at individuals, whereas worry about coronavirus is a stronger predictor of coronavirus policies aimed at individuals. Non-significant interactions suggest that gender, political orientation and general trust in government are all equally strong predictors of support for climate change and coronavirus policies aimed at individuals.

**Table 5:** Support for policies targeting individuals and businesses (Models C2 and C3).

	Individuals		Businesses	
	Model C2 B (95%CI)	Model C3 B (95%CI)	Model C2 B (95%CI)	Model C3 B (95%CI)
*Fixed effects*				
Constant	3.788^***^ (3.739, 3.837)	3.811^***^ (3.758, 3.863)	3.867^***^ (3.820, 3.914)	3.895^***^ (3.846, 3.945)
Climate change (reference: Coronavirus)	−0.296^***^ (−0.331, −0.261)	−0.341^***^ (−0.393, −0.288)	−0.100^***^ (−0.130, −0.070)	−0.156^***^ (−0.201, −0.112)
Female (reference: Male)	0.029 (−0.033, 0.091)	0.032 (−0.040, 0.104)	0.040 (−0.020, 0.101)	0.020 (−0.048, 0.088)
Age	0.036^***^ (0.016, 0.056)	0.066^***^ (0.043, 0.089)	0.046^***^ (0.027, 0.066)	0.074^***^ (0.052, 0.096)
Political orientation	−0.078^***^ (−0.113, −0.043)	−0.095^***^ (−0.135, −0.055)	−0.080^***^ (−0.114, −0.046)	−0.095^***^ (−0.133, −0.057)
General trust in government	0.048^***^ (0.014, 0.081)	0.067^***^ (0.028, 0.106)	0.015 (−0.018, 0.047)	0.047^**^ (0.011, 0.084)
Climate change worry	0.249^***^ (0.220, 0.278)	0.131^***^ (0.097, 0.164)	0.238^***^ (0.209, 0.266)	0.126^***^ (0.094, 0.158)
Coronavirus worry	0.159^***^ (0.130, 0.189)	0.243^***^ (0.209, 0.277)	0.134^***^ (0.105, 0.162)	0.227^***^ (0.195, 0.260)
Climate change × Gender		−0.006 (−0.078, 0.066)		0.040 (−0.021, 0.102)
Climate change × Age		−0.060^***^ (−0.083, −0.037)		−0.055^***^ (−0.075, −0.036)
Climate change × Political orientation		0.034 (−0.007, 0.074)		0.030 (−0.004, 0.065)
Climate change × General trust in government		−0.038 (−0.077, 0.0003)		−0.065^***^ (−0.098, −0.032)
Climate change × Climate change worry		0.237^***^ (0.203, 0.270)		0.223^***^ (0.195, 0.252)
Climate change × Coronavirus worry		−0.168^***^ (−0.202, −0.134)		−0.188^***^ (−0.217, −0.159)
*Random effects*	σ2 (SD)	σ2 (SD)	σ2 (SD)	σ2 (SD)
Level 2 (individual)	0.234 (0.484)	0.237 (0.487)	0.247 (0.497)	0.250 (0.500)
Level 1 (measure)	0.968 (0.984)	0.949 (0.918)	0.715 (0.846)	0.690 (0.831)
*Model fit*				
AIC	35,416	35,164	32,145	31,783
BIC	35,490	35,282	322,219	31,901

Analyses based on 12,040 observations within 1,505 individuals.

*** *p* < 0.01.

There were also a number of significant interactions for policies aimed at businesses (Model C3), showing that age and worry about coronavirus are weaker predictors of support for climate change policies than of support for coronavirus policies (see Table 5). Worry about climate change was a stronger predictor of support for climate change policies than of support for coronavirus policies aimed at businesses. The results further suggest that, while general trust in government may be a significant predictor of support for coronavirus policies aimed at businesses, it is not for climate change policies aimed at businesses. Non-significant interactions suggest that gender and political orientation are both equally strong predictors of support for climate change and coronavirus policies aimed at businesses.

Further detailed analyses (Models C4) were conducted to explore individual differences in support for the specific policy types (see supporting information, Supplementary Table SI6). These show that limiting activities, fining, and financial support are all less supported than informational policies. They further show that limiting individual activity is the least supported policy overall, followed by financial support to encourage people to follow coronavirus regulations, and fining businesses that do not uphold coronavirus regulations. Overall, limiting individual and business activity are the least supported climate change and coronavirus policies. Older age groups are relatively less supportive of financial support, whether to address climate change or coronavirus or whether aimed at individuals or businesses. More right-wing respondents were particularly less supportive of any type of coronavirus policy aimed at businesses. Respondents with a high level of government trust were generally more supportive of the less popular policies of limiting activity and fining, except for those climate change policies that are aimed at businesses. Respondents worried about climate change were also more supportive of the less popular policies of limiting activity and fining, as were those worried about coronavirus.

## Discussion

While parallels have been drawn between the coronavirus outbreak and climate change in previous research, there is limited empirical research directly comparing public responses to the two issues to find out what may and may not be possible in terms of public engagement and action on climate change. This research examined similarities and differences in UK public perceptions on a number of key dimensions, including perceptions of responsibility, the perceived effectiveness of individual action and trust in government, and the acceptability of different types of policies.

The study shows that there are a number of similarities in public responses to coronavirus and climate change. However, major differences exist relating to how the public perceives their own role, i.e., their responsibility to act, their ability to have an impact, and relatedly, their support for policies aimed at curbing individual actions. The results suggest that people feel more personally responsible and think their actions are more efficacious to address the coronavirus outbreak than climate change. Crucially, while people perceive themselves and the government equally responsible for stopping the spread of the coronavirus, they perceive greater government than personal responsibility to try to prevent climate change from worsening. Furthermore, whereas people think that their own personal actions are highly effective in preventing coronavirus from spreading, they are less likely to think that they themselves can help prevent climate change. Interestingly, there was lower trust in the government to act effectively on coronavirus than to act effectively on climate change, possibly reflecting dissatisfaction with the government’s handling of the crisis at the time of data collection (Fancourt et al., 2020).

These findings support and extend prior research showing that the British public feel that the UK government holds the main responsibility (along with big polluters) for tackling climate change (Wang et al., 2020). The public framing of coronavirus may further have facilitated a stronger understanding of the link between people’s actions and the spread of coronavirus, with an emphasis on people having individual responsibility to act, whereas the lower sense of personal responsibility to mitigate climate change reflects previous research findings (Fisher et al., 2018; Bostrom et al., 2020). Overall, this indicates that there may be barriers to engaging people with personal lifestyle changes to address climate change compared to their acceptance of government action. In line with Capstick et al. (2021), we suggest that emphasising the relationship between personal behaviour and wider system change may provide a way for communication and public engagement around climate change to help people understand why individual actions are also important.

Differences in policy support for containing coronavirus and climate change also appear to relate to those focusing on individuals. The lowest levels of support are for limiting individual activity and fining individuals who do not uphold regulation, but then only for climate change. While there is support for these policies to prevent the coronavirus from spreading, the public are less willing to support them to reduce the risk of climate change. As people reported higher personal responsibility and efficacy in relation to coronavirus, it is perhaps unsurprising that they were also more willing to support policies targeting individuals for this purpose. It however has to be noted that, while support for climate change policies was generally lower than for coronavirus policies, it was on average still above the midpoint for all policies included. This suggests there is no strong opposition to what perhaps can be considered radical policies to address climate change. The results from our research appear to largely reflect previous findings. They confirm that policies targeting individuals are seen as less acceptable than those targeting businesses (Swim and Geiger, 2021). In particular, measures that restrict personal behaviours, such as flying or eating meat (Kantenbacher et al., 2018; Ash and Zimmermann, 2020), or those that use financial disincentives, such as fines and taxes to discourage them (de Groot and Schuitema, 2012; Drews and van den Bergh, 2016; Swim and Geiger, 2021), are generally unpopular. On the other hand, financial incentives to help individuals comply with climate change regulations were relatively supported, again in line with existing research (Swim and Geiger, 2021).

The study further examined the role of individual-level variables in coronavirus and climate change related perceptions and policy support. Across both issues, women felt a greater personal responsibility and higher personal efficacy to act than men. This reflects prior research showing that women feel a higher sense of personal responsibility towards tackling climate change (Boto-García and Bucciol, 2020) and are more likely than men to think their own personal actions can help to address climate change (Department for Business, Energy and Industrial Strategy, 2021). However, gender was not a significant predictor of perceived government responsibility, government trust, or policy support for the two issues; showing that gender differences are for views on individual action only.

Participants’ age seems to be a key demographic variable that differentiates people’s responses to coronavirus and climate change. Older respondents felt a greater personal responsibility and a higher personal efficacy in relation to the coronavirus, but this effect was weaker or even reversed for climate change. Similarly, older respondents ascribed higher levels of responsibility to the government in relation to coronavirus, but this effect was weaker for climate change. Furthermore, older respondents were more supportive of coronavirus policies targeting either individuals or businesses. These effects were weaker for climate change. Given that coronavirus appears to pose a greater health risk for older people (World Health Organization, 2020), it may currently have more salience for them than climate change, despite similar levels of agreement across generations that significant lifestyle changes are needed to address climate change (Duffy, 2021).

Political orientation is a further key factor in coronavirus and climate change-related perceptions and policy support. Across the two issues, right-wing respondents reported lower personal and government responsibility; and they expressed lower levels of support for policies targeting both individuals and businesses. Crucially, the political orientation effects were the same for coronavirus and climate change, suggesting that they reflect general opinions on public policy rather than specific views on how to deal with the two issues (e.g., Dimock et al., 2014). This is in line with other research showing consistent differences between left-wing and right-wing individuals in their perceptions and policy support relating to coronavirus (Ramos et al., 2020; Ju and You, 2021) and climate change (Drews and van den Bergh, 2016; Fisher et al., 2018; Hornsey et al., 2021).

Government trust was further identified as a driver of coronavirus and climate change related perceptions and policy support. Importantly, government trust was associated with higher levels of perceived personal responsibility and willingness to support individual-level policies, suggesting that people’s willingness to act (and accept government action) on the two issues is part of a social contract between citizens and the state (Howarth et al., 2020). The results highlight the importance of public trust in government and its institutions during global crises, such as the coronavirus outbreak (Wright et al., 2020; Pak et al., 2021) and climate change (Fairbrother et al., 2019; Kulin and Johansson Sevä, 2021; Bergquist et al., 2022).

The most important factor driving public perceptions and policy support was, however, worry about the two issues. Coronavirus and climate change worry were among the strongest individual-level predictors across all outcome variables. They were associated with higher levels of perceived personal responsibility and efficacy, as well as higher levels of perceived government responsibility and trust. Individuals worried about coronavirus and climate change were more supportive of policies to address the two issues. In particular, they were more willing to support less popular measures that limit individual activity or use financial disincentives (i.e., fines) to enforce compliance. These results most likely reflect the greater motivation they have to see the risks of the two issues reduced. However, as the coronavirus pandemic has had negative impacts on mental health and wellbeing in the UK, including anxiety, (Office for Health Improvement and Disparities, 2021) this could have influenced how people felt about other issues such as climate change.

As with any research, there are a number of caveats that should be considered when interpreting the results. The research was limited to the UK context and therefore the findings may not be applicable to other countries, particularly where coronavirus and climate change are being dealt with differently or have had different impacts. Nevertheless, as both coronavirus and climate change continue to be issues facing all countries in the world, research examining public responses in the UK context may still provide useful comparison and insights, particularly around public perceptions of risk and efficacy regarding the two issues, and how that relates to the kinds of policies the public are willing to support. This study has implications both for addressing a gap in the literature as well as broader impacts for how policymakers can respond to and frame coronavirus and climate change.

The results must also be contextualised in the temporal context of the survey, which was conducted in November and December 2020, at the start of the second wave of the coronavirus pandemic, when, in the UK government’s own words, the virus was “out of control” (BBC, 2020), and the UK was facing a second lockdown. It was also largely before the roll out of the vaccination programme, which started on 8 December 2020 in the UK. This may have impacted the responses, in particular regarding the government. There may therefore also have been differences in the salience of the two issues, with people being directly confronted with the consequences of the pandemic and policy responses on a day-to-day basis, and virtually continuous media coverage at the time of the study. This may have instilled a greater sense of urgency to deal with the issue as expressed in higher levels of policy support; although other work has found little evidence to support the hypothesis that a “finite pool of worry” has diminishing concern about climate change (Evensen et al., 2021). Steentjes et al. (2021) showed that people’s concern and sense of urgency about climate change remained high during the pandemic.

There may however have been more subtle framing effects that underlie the results. Throughout the pandemic there was coordinated crisis communication alongside regular press conferences. An analysis of the UK government discourse during the first nine months of the pandemic showed a prominent theme of shared responsibility, but at the same time placing the responsibility for the spread and thus containment of the virus on individuals (Andreouli and Brice, 2021). The findings of the research could be contextualised even further. While the study found that the main differences relate to individual action, that does not mean that climate inaction is due to these individual factors (Schmitt et al., 2020). It however does show that people feel a lack of agency to take action. In many cases, individuals are limited in the types of action they can take to address a global systemic threat (Uzzell and Räthzel, 2009). The scale of the challenge to address climate change requires both system and individual change (Capstick et al., 2021). This however needs a careful consideration of the factors that can be addressed at the individual level and those that are beyond individual control and the responsibility for institutions and businesses (Akenji et al., 2021).

Since the research was conducted, the UK has experienced multiple coronavirus outbreaks (including the Delta and Omicron variants), rolled out multiple (booster) vaccination programmes, and imposed different levels of restrictions (with differences between the four UK home nations). In addition, revelations of government staff and others breaking lockdown restrictions (‘partygate’) may have had a profound impact on trust in government and as a result the public’s willingness to accept and adhere to measures, not only to deal with the coronavirus pandemic but also with other policy areas; although this view is contested by some academics (Reicher, 2021). It would therefore be useful to repeat the research to assess how the different aspects of coronavirus related perceptions have changed over the course of the pandemic, and their implications for support for policies to deal with other collective risks, such as climate change.

Another caveat relates to the results for support for coronavirus and climate change policies. While great care was taken to formulate similar types of policy for coronavirus and climate change, the scale of these policy measures may still be perceived to be different. For example, support for restrictions on air travel to contain coronavirus or climate change may differ because those to contain coronavirus are seen as time limited and more effective (Kallbekken and Sælen, 2021). The difference in attitudes to policies that limit personal freedom may therefore reflect the different timescales over which such restrictions would apply: the assumption being they would be more long-term, or even permanent, for climate change. It was beyond the scope of this study to explore what perceptual and contextual factors may underlie the differences between, for example, support for coronavirus and climate change support. The conditions under which climate change policies are acceptable to the wider public is however an important avenue for future research.

A methodological caveat relates to potential order effects where responses to the two topics may be influenced by the order in which the questions are presented. The presentation of the first topic (e.g., climate change) may frame the responses to the subsequent topic (e.g., coronavirus). Similarly, support for the different policies may be framed by the presentation and response to the first policy that is shown from the list. In order to control for these order effects, the coronavirus and climate change questions were counter balanced and the order of policies randomised. The different measures (e.g., responsibility, efficacy, and trust) were presented in a fixed order, which may have produced subtle order effects to the measure responses. However, the main comparison was between coronavirus and climate change which is unlikely to have been influenced by this. Great care was taken to align the phrasing of the questions, not only across the two topics but also across the different measures to ensure responses to them can be compared.

## Conclusion

This study directly compared public responses to coronavirus and climate change in the UK, comparing similarities and differences between how people saw the two issues, and their own role in containing the crises. Our analysis showed differences in how the public perceives their own role in addressing coronavirus and climate change: their responsibility to act and their ability to have an impact as well as the government’s role. People see themselves as less responsible and their actions as less effective at stopping climate change, and there is lower support for more far-reaching climate change policies that target individuals. The findings may relate to there being a greater emphasis on clear, concrete actions explaining how individuals can help to contain the coronavirus in government and wider public health communications, while the same cannot be said for climate change. Future research could assess how the different aspects of coronavirus related perceptions changed during the pandemic and what implications this has for support for climate change policies, as well as exploring what perceptual and contextual factors may underlie the differences between support for coronavirus and climate change support.

These findings suggest that experiences from the coronavirus pandemic cannot directly be translated to climate change, and thus that climate change is likely to require different policy responses and framing. Although individual action should not be emphasised at the expense of system change, as both are needed to tackle climate change, our findings point to a role for public engagement to make a clearer link between the two.

## Data availability statement

All data and materials supporting this study are openly available on https://osf.io/2x3m6 (doi.org/10.17605/OSF.IO/2X3M6).

## Ethics statement

The studies involving human participants were reviewed and approved by School of Psychology, Cardiff University. The participants provided their written informed consent to participate in this study.

## Author contributions

WP and SW: formal analysis, writing – original draft, review and editing, conceptualization, and methodology. BL: writing – original draft, and review and editing. All authors contributed to the article and approved the submitted version.

## Funding

This project received support from the Economic & Social Research Council (ESRC) through the Centre for Climate Change & Social Transformations (CAST), Grant Ref: ES/S012257/1. The project was funded through the CAST Impact Fund.

## Conflict of interest

The authors declare that the research was conducted in the absence of any commercial or financial relationships that could be construed as a potential conflict of interest.

## Publisher’s note

All claims expressed in this article are solely those of the authors and do not necessarily represent those of their affiliated organizations, or those of the publisher, the editors and the reviewers. Any product that may be evaluated in this article, or claim that may be made by its manufacturer, is not guaranteed or endorsed by the publisher.

## Footnotes

^1^An advisory board consisting of seven academics convened at the project inception and during the design phase of the survey. A stakeholder panel (*N* = 8), selected through purposeful sampling, asked experts (who, in their work, represent and advocate for the interests of marginalised groups) to provide an informed view of how different audiences in the UK may have experienced COVID-19. Two online one-hour community panels (*N* = 8) were conducted through a community research panel, to sensitise the research team on how COVID-19 had affected the public’s feelings towards climate change.
