# Supplementary material for: Comparing coronavirus (COVID-19) and climate change perceptions: Implications for support for individual and collective-level policies
Source: Front Psychol. 2022 Oct 5;13:996546. doi: 10.3389/fpsyg.2022.996546 (PMC9580362; doi:10.3389/fpsyg.2022.996546)
Supplement: Supplementary file 1 [file Table_1.docx]

Supplementary Material

# Supporting Information

**Table SI1. Perceived personal and government responsibility (Figure 1) and Perceived efficacy and trust (Figure 2)**

|  | Coronavirus  M (SD) | Climate change  M (SD) |
| --- | --- | --- |
| Perceived personal responsibility | 8.01 (2.33) | 6.41 (2.67) |
| Perceived government responsibility | 8.26 (2.10) | 7.81 (2.39) |
| Perceived personal efficacy | 7.90 (2.39) | 5.86 (2.78) |
| Government trust | 5.27 (3.02) | 5.94 (2.78) |

| **Table SI2. Perceived personal and government responsibility (Model A1)** | | |
| --- | --- | --- |
|  | **Perceived responsibility** | |
|  | **Step 1**  **B (95%CI)** | **Step 2**  **B (95% CI)** |
| Constant | 7.726^***^ (7.614, 7.838) | 8.014^***^ (7.895, 8.134) |
| Climate change (reference: Coronavirus) | -1.030^***^ (-1.119, -0.941) | -1.607^***^ (-1.731, -1.484) |
| Government responsibility (reference: Personal responsibility) | 0.820^***^ (0.731, 0.909) | 0.242^***^ (0.119, 0.366) |
| Climate change x Government responsibility |  | 1.155^***^ (0.981, 1.330) |
| *Model Fit*  AIC  BIC | 26400  26434 | 26237  26278 |
| *Note:* Analysis based on 6,072 observations within 1,518 individuals; ^**^p<0.05; ^***^p<0.01 | | |

| **Table SI3. Perceived personal efficacy and government trust (Model B1)** | | |
| --- | --- | --- |
|  | **Personal efficacy and Government trust** | |
|  | **Step 1**  **B (95%CI)** | **Step 2**  **B (95% CI)** |
| Constant | 7.222^***^ (7.092, 7.352) | 7.902^***^ (7.763, 8.040) |
| Climate change (reference: Coronavirus) | -0.682^***^ (-0.800, -0.564) | -2.042^***^ (-2.199, -1.884) |
| Trust (reference personal efficacy) | -1.275^***^ (-1.393, -1.157) | -2.634^***^ (-2.792, -2.477) |
| Climate change x Trust |  | 2.719^***^ (2.496, 2.942) |
| *Model Fit*  AIC  BIC | 29193  29226 | 28656  28696 |
| *Note:* Analysis based on 6,072 observations within 1,518 individuals; ^**^p<0.05; ^***^p<0.01 | | |

**Table SI4. Mean (M) and standard deviation (DV) of support for policies to address coronavirus and climate change.**

|  | **Coronavirus** | | **Climate change** | |
| --- | --- | --- | --- | --- |
|  | **Individuals**  **M (SD)** | **Businesses**  **M (SD)** | **Individuals**  **M (SD)** | **Businesses**  **M (SD** |
| Limiting activity | 3.93 (1.19) | 3.55 (1.21) | 3.16 (1.25) | 3.74 (1.06) |
| Fining | 4.08 (1.11) | 4.17 (1.08) | 3.57 (1.19) | 4.00 (1.07) |
| Financial support | 3.46 (1.23) | 4.08 (0.94) | 3.74 (1.07) | 3.78 (0.99) |
| Information provision | 4.41 (0.86) | 4.37 (0.86) | 4.22 (0.93) | 4.25 (0.91) |

| **Table SI5. Support for coronavirus and climate change policies targeting individuals or businesses (Model C1)** | | |
| --- | --- | --- |
|  | **Policy support** | |
|  | **Step 1**  **B (95%CI)** | **Step 2**  **B (95% CI)** |
| Constant | 4.089^***^ (4.051, 4.127) | 4.040^***^ (4.000, 4.080) |
| Climate change (reference: Coronavirus) | -0.197^***^ (-0.220, -0.175) | -0.099^***^ (-0.131, -0.067) |
| Individual (reference: Business) | -0.169^***^ (-0.192, -0.147) | -0.071^***^ (-0.103, -0.039) |
| Climate change x Individual |  | -0.197^***^ (-0.243, -0.151) |
| *Model Fit*  AIC  BIC | 67591  67631 | 67521  67570 |
| *Note: Analysis based on* 24,288 observations within 1,518 individuals; ^**^p<0.05; ^***^p<0.01. | | |

| **Table SI6. Support for coronavirus and climate change policies targeting individuals or businesses (Model C4)** | | | | |
| --- | --- | --- | --- | --- |
|  | **Individuals** | | **Businesses** | |
|  | **Climate change** | **Coronavirus** | **Climate change** | **Coronavirus** |
|  | **B (95%CI)** | **B (95%CI)** | **B (95%CI)** | **B (95%CI)** |
| Constant | 4.018^***^ (3.941, 4.095) | 4.302^***^ (4.224, 4.380) | 4.033^***^ (3.964, 4.101) | 4.261^***^ (4.188, 4.333) |
| Policy type: Limit activity^(a)^ | -1.083^***^ (-1.174, -0.992) | -0.547^***^ (-0.637, -0.457) | -0.520^***^ (-0.593, -0.446) | -0.845^***^ (-0.924, -0.766) |
| Policy type: Fining^(a)^ | -0.654^***^ (-0.745, -0.563) | -0.342^***^ (-0.432, -0.252) | -0.239^***^ (-0.312, -0.165) | -0.218^***^ (-0.297, -0.139) |
| Policy type: Financial support^(a)^ | -0.421^***^ (-0.512, -0.330) | -0.946^***^ (-1.037, -0.856) | -0.398^***^ (-0.471, -0.324) | -0.274^***^ (-0.353, -0.195) |
| Female (reference: Male) | 0.079 (-0.027, 0.184) | 0.090 (-0.018, 0.198) | 0.124^***^ (0.030, 0.218) | 0.093 (-0.008, 0.194) |
| Age | 0.045^***^ (0.011, 0.079) | 0.113^***^ (0.079, 0.148) | 0.051^***^ (0.020, 0.081) | 0.104^***^ (0.072, 0.137) |
| Political orientation | -0.033 (-0.091, 0.026) | -0.104^***^ (-0.164, -0.045) | -0.047^*^ (-0.100, 0.005) | -0.060^**^ (-0.115, -0.005) |
| General trust in government | -0.007 (-0.064, 0.050) | 0.009 (-0.050, 0.067) | -0.003 (-0.053, 0.048) | -0.0003 (-0.055, 0.054) |
| Climate change worry | 0.365^***^ (0.319, 0.411) | --- | 0.335^***^ (0.295, 0.376) | --- |
| Coronavirus worry | --- | 0.164^***^ (0.117, 0.212) | --- | 0.177^***^ (0.132, 0.221) |
| Limit activity x Female | 0.005 (-0.120, 0.129) | 0.003 (-0.122, 0.128) | -0.036 (-0.136, 0.065) | -0.117^**^ (-0.226, -0.007) |
| Fining x Female | -0.084 (-0.209, 0.041) | -0.091 (-0.216, 0.033) | -0.091 (-0.192, 0.010) | -0.072 (-0.181, 0.037) |
| Financial support x Female | -0.097 (-0.221, 0.028) | -0.074 (-0.199, 0.051) | -0.108^**^ (-0.209, -0.007) | -0.035 (-0.144, 0.075) |
| Limit activity x Age | -0.019 (-0.059, 0.021) | -0.025 (-0.066, 0.015) | -0.027 (-0.060, 0.005) | -0.069^***^ (-0.104, -0.033) |
| Fining x Age | -0.034 (-0.075, 0.006) | -0.002 (-0.043, 0.038) | 0.003 (-0.029, 0.036) | 0.014 (-0.021, 0.049) |
| Financial support x Age | -0.097^***^ (-0.138, -0.057) | -0.176^***^ (-0.216, -0.136) | -0.102^***^ (-0.135, -0.070) | -0.081^***^ (-0.116, -0.046) |
| Limit activity x Political orientation | -0.047 (-0.117, 0.022) | -0.069^**^ (-0.138, -0.001) | 0.003 (-0.053, 0.060) | -0.098^***^ (-0.158, -0.038) |
| Fining x Political orientation | -0.012 (-0.081, 0.058) | 0.006 (-0.062, 0.075) | -0.051 (-0.107, 0.006) | -0.078^**^ (-0.138, -0.018) |
| Financial support x Political orientation | -0.038 (-0.108, 0.031) | -0.021 (-0.090, 0.047) | -0.012 (-0.069, 0.044) | -0.081^***^ (-0.142, -0.021) |
| Limit activity x Government trust | 0.082^**^ (0.015, 0.150) | 0.109^***^ (0.041, 0.176) | -0.037 (-0.092, 0.017) | 0.071^**^ (0.012, 0.130) |
| Fining x Government trust | 0.084^**^ (0.016, 0.151) | 0.127^***^ (0.059, 0.194) | -0.009 (-0.063, 0.045) | 0.096^***^ (0.037, 0.155) |
| Financial support x Government trust | -0.008 (-0.075, 0.059) | -0.019 (-0.087, 0.048) | -0.006 (-0.061, 0.048) | 0.008 (-0.051, 0.067) |
| Limit activity x Climate change worry | 0.041 (-0.014, 0.095) | --- | 0.070^***^ (0.026, 0.113) | --- |
| Fining x Climate change worry | 0.103^***^ (0.049, 0.157) | --- | 0.085^***^ (0.041, 0.128) | --- |
| Financial support x Climate change worry | -0.023 (-0.078, 0.031) | --- | -0.039 (-0.083, 0.005) | --- |
| Limit activity x Coronavirus worry | --- | 0.197^***^ (0.142, 0.252) | --- | 0.231^***^ (0.183, 0.280) |
| Fining x Coronavirus worry | --- | 0.177^***^ (0.122, 0.232) | --- | 0.151^***^ (0.103, 0.200) |
| Financial support x Coronavirus worry | --- | 0.141^***^ (0.085, 0.196) | --- | 0.013 (-0.035, 0.062) |
| *Model Fit*  AIC  BIC | 16601  16776 | 16773  16947 | 14601  14775 | 15528  15702 |
| *Note: Analysis based on 6,020 observations within 1,502 individuals*; *^**^p<0.05; ^***^p<0.01; ^(a)^reference: information.* | | | | |
